# Transcriptome-Guided Drug Repurposing Identifies Homoharringtonine (HHT) as a Candidate for Radiation-Induced Pulmonary Fibrosis

**DOI:** 10.3390/pharmaceutics17121626

**Published:** 2025-12-18

**Authors:** Mohamed El-Agamy Farh, Sang Yeon Kim, Sunjoo Park, Cui Ronglan, InSuk Sohn, Jaeho Cho

**Affiliations:** 1 Department of Radiation Oncology, College of Medicine, Yonsei University, Seoul 03722, Republic of Korea; mafarh@ssu.ac.kr (M.E.-A.F.); sysykim@honam.ac.kr (S.Y.K.); sunjoo77sun@hotmail.com (S.P.); choiyr322@yuhs.ac (C.R.); 2 Drug Development Team, ARONTIER Inc., Seoul 06735, Republic of Korea; issohn@arontier.co; 3 AI-Bio Convergence Research Institute, Soongsil University, Seoul 06978, Republic of Korea; 4 Department of Food and Nutrition, College of Health Science, Honam University, Gwangju 62399, Republic of Korea

**Keywords:** radiation-induced pulmonary fibrosis, drug repurposing, LINCS, REMEDY, homoharringtonine, RhoA/ROCK, Wnt/β-catenin

## Abstract

**Background:** Radiation-induced pulmonary fibrosis (RPF) remains a major burden of successful lung cancer radiotherapy. Clinically validated drugs targeting RPF remains scarce. **Methods:** We employed a transcriptome-based drug repurposing approach using REMEDY, a computational platform built on the Library of Integrated Network-Based Cellular Signatures (LINCS). Differentially expressed genes (DEGs) derived from radiation-induced lung injury (RILI) models were used as a query to identify compounds capable of reversing pro-fibrotic expression profile. Among top-ranked candidates, homoharringtonine (HHT), an FDA-approved protein synthesis inhibitor, was selected for experimental validation. Anti-fibrotic effects of HHT were assessed using an optimized in vitro fibrotic model based on activation of MRC-5 human lung fibroblasts. Complementary in silico molecular docking analyses were also conducted to explore the mechanistic basis of HHT’s actions. This represents the first transcriptome-guided, LINCS-based drug repurposing study applied specifically to radiation-induced pulmonary fibrosis, utilizing RPF-derived molecular signatures rather than general fibrosis-related datasets. **Results:** HHT significantly attenuated key fibrotic phenotypes, including fibroblast proliferation, myofibroblast differentiation, and extracellular matrix (ECM) production. Notably, HHT suppressed expression of cyclin D1 and α-smooth muscle actin (α-SMA), and reduced collagen deposition. Mechanistic investigations revealed that HHT modulates two pro-fibrotic pathways: RhoA/ROCK and Wnt/β-catenin signaling. Molecular docking further suggested that HHT may directly interact with fibrosis-related receptors such as integrins and Frizzled, providing structural insight into its anti-fibrotic potential. These findings underscore the novelty of reassigning HHT to a radiation-specific fibrotic context using a signature-reversal strategy uniquely tailored to RPF biology. **Conclusions:** Our findings identify HHT as a promising treatment of RPF, offering a dual mechanism of action—interruption of protein synthesis and targeted inhibition of fibrotic signaling pathways. This study highlights the value of computational drug repurposing platforms for accelerating therapeutic discovery. Further preclinical investigations are warranted to evaluate HHT’s in vivo efficacy and clinical applicability in RPF.

## 1. Introduction

Modern radiotherapy has revolutionized the management of lung cancer, offering effective treatment options across all disease stages, either as standalone or in combination with other modalities [1]. However, the therapeutic benefits of radiotherapy are often offset by its dose-limiting toxicities, particularly pneumonitis and fibrosis, resulting from collateral damage to surrounding healthy lung tissues [2]. The ionizing radiation (IR) inflicts direct damage to alveolar epithelial cells—primarily type 1 pneumocytes—eliciting a persistent inflammatory response characterized by immune cell recruitment and cytokine release at the injury site. In many instances, this unresolved inflammation progresses into irreversible scarring, clinically manifested as radiation-induced pulmonary fibrosis (RPF) [3].

RPF is a progressive and debilitating late toxicity that can occur after thoracic radiotherapy. It is driven by dysregulated immune signaling and chronic overexpression of profibrotic mediators such as transforming growth factor-β1 (TGF-β1), tumor necrosis factor-α (TNF-α), interleukin-1β (IL-1β), and interleukin-6 (IL-6) [4]. These factors trigger epithelial–mesenchymal transition (EMT), excessive extracellular matrix (ECM) deposition, fibroblast-to-myofibroblast differentiation, and progressive tissue stiffening [5]. RPF remains a major clinical burden, as its incidence increases with dose-escalated radiotherapy and modern combined-modality treatment strategies. Importantly, no FDA-approved pharmacologic therapy currently exists for preventing or treating RPF, underscoring the need for novel therapeutic approaches.

Although RPF shares several pathological features with idiopathic pulmonary fibrosis (IPF)—including myofibroblast activation, ECM accumulation, and persistent inflammatory signaling—it differs fundamentally in its etiology, temporal dynamics, and transcriptomic architecture. RPF is initiated by ionizing-radiation–induced DNA damage and sterile inflammation, whereas IPF arises from repetitive microinjury and aberrant wound-repair processes of unknown origin. These distinctions highlight the importance of studying RPF as a biologically unique disease entity and justify the use of RPF-specific transcriptomic signatures for therapeutic discovery. Leveraging these disease-specific molecular profiles may enable more precise drug-repurposing strategies tailored to radiation-triggered fibrotic remodeling.

Despite advances in the understanding of pulmonary fibrosis, treatment options remain limited. To date, only two FDA-approved drugs-pirfenidone (Esbriet) and nintendanib (Ofev)-are available for fibrotic lung conditions [6,7,8,9]. Both agents act by inhibiting fibroblast proliferation and ECM production, and by modulating signaling cascades involved in fibrotic remodeling. However, their efficacy in RPF remains suboptimal, and their broad adoption is hampered by side effects and high costs.

Given the high attrition rates, extended timelines, and escalating costs associated with de novo drug discovery [10], drug repurposing has emerged as a compelling strategy to expedite the development of new therapies for unmet medical needs [11,12]. This approach leverages large-scale transcriptomic databases, such as the Connectivity Map (CMap) and the Library of Integrated Network-Based Cellular Signatures (LINCS), which catalog drug-induced gene expression changes across diverse cell types and treatment conditions [13,14,15].

Central to this strategy is the concept of “signature reversion,” wherein compounds that reverse disease-associated gene expression patterns are considered potential therapeutics [13]. This methodology has been successfully applied in several disease domains, including cancer [16,17], muscle atrophy [18], acute myeloid leukemia [19], and neurodegenerative diseases such as Parkinson’s [20].

In this study, we utilized REMEDY, a proprietary LINCS-based drug repurposing platform developed by Arontier Co. (Seoul, Republic of Korea), to identify candidate therapeutics for RPF. REMEDY integrates advanced pattern-matching algorithms and gene signature enrichment to systematically predict compounds that may reverse fibrotic transcriptomic profiles. Using differentially expressed genes (DEGs) from an established radiation-induced lung injury (RILI) model, our screening highlighted homoharringtonine (HHT), an FDA-approved alkaloid used in leukemia treatment, as a top-ranked candidate. HHT was prioritized not only based on its high negative connectivity score but also due to emerging evidence of its anti-fibrotic properties in other disease contexts. We proceeded to experimentally validate HHT in an in vitro model of RPF and investigated its mechanistic actions on key fibrosis-related signaling pathways. Although HHT has demonstrated anti-fibrotic activity in other organ systems, radiation-induced pulmonary fibrosis (RPF) arises from distinctly different initiating events—including DNA damage–driven epithelial injury, radiation-specific cytokine dynamics, and unique transcriptomic remodeling. Importantly, our study is the first to apply a LINCS/CMap-based reverse gene-signature approach to RPF-specific molecular profiles derived from an established radiation-induced lung injury model. By leveraging these radiation-context signatures rather than generic fibrosis datasets, we identified HHT as a top-ranked reverse-signature compound. This novelty lies in the disease context and the transcriptome-guided discovery framework rather than in proposing an entirely new molecular mechanism for HHT.

## 2. Materials and Methods

### 2.1. Reagent

HHT was purchased from Sigma-Aldrich (St. Louis, MO, USA). The compound was dissolved in dimethyl sulfoxide (DMSO), stored at 4 °C, and diluted to the required working concentrations immediately before use.

### 2.2. Cell Line and Radiation Treatment

MRC-5, a lung fibroblast isolated from the lung tissue of a white male, 14-week-old embryo, were purchased from Korean Cell Line Bank (KCLB number: 10771). Cells were cultured in Minimal Essential Media (MEM; WELGENE, Gyeongsangbuk-do, Republic of Korea) supplied with 10% heated deactivated fetal bovine serum (FBS; WELGENE, Gyeongsangbuk-do, Republic of Korea) and maintained at 37 °C with 5% of CO_2_. For establishing the in vitro fibrotic model, cells were irradiated with 4 Gy (1.6 Gy/min, 300 kV, 7.86 mA) using X-RAD 320 (Precision, North Branford, CT, USA) and immediately supplied with approximately 5 ng/mL of recombinant human TGF-β1 (PeproTech, Cranbury, NJ, USA).

### 2.3. Differential Gene Expression (DEG) Collection

To identify drug candidates capable of reversing RILI-related gene signatures, previously published DEG datasets from a mouse model of radiation-induced lung injury were retrieved [21]. These datasets included four experimental comparisons categorized by radiation dose (65 Gy and 75 Gy) and pathological phase (inflammatory vs. fibrotic); 65Gy_2W, 65Gy_6W, 75Gy_2W, and 75Gy_6W.

### 2.4. Drug/Target Prediction Using LINCS L1000 Database

Transcriptomic-based drug prediction was conducted using the REMEDY platform, developed by Arontier Inc. REMEDY operates on level 4 data from the LINCS L1000 molecular signature database, comprising over 1.2 million differential gene expression profiles normalized by z-score transformation. These datasets (Phase I: GSE92742; Phase II: GSE70138) were accessed via the Gene Expression Omnibus (GEO). Perturbagen information including Mode of Action (MoA) and clinical development phase was obtained from the Drug Repurposing Hub (https://clue.io/repurposing accessed on 11 June 2024). Pattern-matching was performed using two algorithms: the original CMap Kolmogorov–Smirnov (KS)-based score and a modified KS-dependent score proprietary to Arontier Inc. Only reverse (negative) connectivity scores were considered, as all input signatures were disease-associated. Each compound’s frequency among the top 1000 ranked signatures was normalized and assessed for statistical significance using Fisher’s exact test. Candidates with fewer than four hits or a *p*-value > 0.05 were excluded. Top-ranked perturbagens were selected for experimental validation.

### 2.5. Colony Formation Assay (CFA)

MRC5 cells were seeded in a designated plating cell number in 6-well plates overnight. Then, cells were treated with HHT and IR with 2, 4, 6, and 8 Gy using X-RAD 320 (Precision, North Branford, CT, USA). Cells were then re-cultured in MEM media supplied with 10% FBS at 37 °C with 5% (*v*/*v*) CO_2_ for nearly 2 weeks. Afterwards, cells were washed, fixed with cold methanol for 1 h, and then stained with 2% crystal violet. Colonies were quantified by visual observation of colony number counting. The final DMSO concentration in all treatments, including the 0 nm vehicle control, was ≤0.05% (*v*/*v*).

### 2.6. Scratch Wound Migration Assay

In order to conduct tip-scratch assay, MRC5 cells were seeded in 35 mm plates at 37 °C with 5% (*v*/*v*) CO_2_ for overnight so that cells were allowed to adhere and spread over the plate to create confluent (95–100%) monolayer cells. Then, cells were washed, media were changed with fresh one, and cells monolayer was carefully scratched from the middle using clean and sterilized pipette tip. Then, extend of cell migration was measured by periodically monitoring and measuring the cell-free area under microscope for 24 h. Vehicle exposure was standardized across groups (final DMSO ≤ 0.05%).

### 2.7. Cell Viability Assay

To investigate the cell viability following the treatment, cells were seeded in 96-microwell plates at 37 °C with 5% (*v*/*v*) CO_2_ overnight. Next day, cells were treated and incubated at 37 °C with 5% (*v*/*v*) CO_2_ for 24 h, 48 h, and 72 h. At each harvesting time, cells were treated with cell proliferation reagent WST-1 (Roche Hungary Ltd., Budapest, Hungary) in dark for 2 h. The plates were then read on a microplate spectrophotometer at 450 nm and the viability percentage was calculated by comparing the cell viability following the treated relative to the viability of treatment-free cells. To control for solvent effects, all HHT working dilutions were prepared in sterile water, with a final DMSO concentration ≤ 0.05% (*v*/*v*). The 0 nm group received the same vehicle solution without HHT.

### 2.8. Western Blotting

Cells were lysed in RIPA buffer (50 mMTris-HCl, pH 7.4; 1% Nonidet P-40; 0.25% sodium deoxycholate; 150 mm NaCl; 1 mm Na3VO4) containing protease inhibitors (2 mm phenylmethylsulfonyl fluoride, 100 μg/mL leupeptin, 10 μg/mL pepstatin, 1 μg/mL aprotinin, and 2 mm EDTA) and a phosphatase inhibitor cocktail (GenDEPOT, Baker, TX, USA). After incubation for 30 min, the lysates were centrifuged at 15,000 rpm for 20 min at 4 °C, and the supernatants were obtained for Western blotting. Evaluation of protein concentration was conducted using a BCA protein kit (Bio-Rad, Hercules, CA, USA). Extracted proteins (10 µg) were loaded and separated using sodium dodecyl sulfate–polyacrylamide gel electrophoresis (SDS-PADGE) for 2 h and transferred onto polyvinylidene fluoride membranes (GE Healthcare, Little Chalfont, UK) for 2 h. For membrane blocking, membranes were incubated with 5% skim milk for nearly 2 h at room temperature. Anti-α-SMA (1:3000, Abcam, Cambridge, UK), Anti-cyclin D1 (1:1000; Cell Signalling Technology, Danvers, MA, USA), anti-β catenin (total) (1:500; Cell Signalling Technology), anti-β catenin (active) (1:500; Cell Signalling Technology), anti-ROCK1 (1:000; Cell Signalling Technology), anti-GAPDH (1:2000; Cell Signalling Technology).

### 2.9. Collagen Staining

MRC-5 cells were seeded in 6-well plates and maintained in the abovementioned condition overnight. Then, cells were activated by IR with 4 Gy followed by addition of 5 ng/mL of recombinant human TGF-β1 and then incubated in the same condition for 72 h. Then, cells were washed by PBS, fixed with cold 75% ethanol for 1 h, washed again with PBS, and stained with Direct Red 80 dissolved in picric acid (*w*/*v*) for 1 h. Cells were washed with 0.01 N HCL to remove unbonded stain, and then photographed under microscope for image staining analysis using ImageJ v1.54d.

### 2.10. Molecular Docking

Receptor structures of α4β7 (PDB ID: 3V4V), α5β1 (PDB ID: 4WK0), and Frizzled (FzD; PDB ID: 5UWG) were retrieved from the Protein Data Bank (PDB). All receptor structures were prepared using AutoDock Tools 1.5.7 [22] by removing crystallographic water molecules, adding polar hydrogens, computing Gasteiger partial charges, and converting the files into PDBQT format. The ligand HHT was obtained from PubChem, energy-minimized using the MMFF94 force field, and converted to PDBQT format using Open Babel [23].

Molecular docking was performed using AutoDock Vina 1.2.0 [24]. Grid boxes were positioned to cover the experimentally validated or predicted ligand-binding regions of each receptor, with grid centers defined as follows: α4β7 (x = −15.96 Å, y = 14.78 Å, z = 39.36 Å), α5β1 (x = 21.04 Å, y = 11.78 Å, z = −20.64 Å), and FzD (x = 4.00 Å, y = 109.00 Å, z = −9.00 Å) and dimensions size as follows: α4β7 (44 × 52 × 46 points), α5β1 (40 × 40 × 40 points), and FzD (51 × 51 × 51 points). A total of 10 binding poses were generated per docking run using an exhaustiveness of 16. Grid box dimensions were selected to ensure full coverage of the binding pocket while minimizing inclusion of irrelevant surface regions.

To validate the reliability of the docking protocol, each receptor’s co-crystallized ligand was redocked, yielding RMSD values ≤ 2.0 Å between native and redocked poses, confirming the robustness of the docking parameters. Binding significance was evaluated based on three criteria: (1) a predicted binding energy (ΔG) ≤ −6.0 kcal/mol or within 1.0 kcal/mol of the reference ligand for that receptor; (2) the presence of conserved interactions with key amino acids known from SAR or crystallographic studies; and (3) consistency of the docking pose with validated binding-site geometry.

Binding affinities, residue-level interaction patterns, and 2D/3D ligand contact maps were analyzed using Flare Viewer v10. All receptor–ligand structures, docking parameters, and grid definitions were documented to ensure methodological transparency and reproducibility.

### 2.11. Reproducibility and Replicates

All experiments were performed using three independent biological replicates, each derived from separately cultured cell populations prepared on different days. For viability assays (WST-1), colony-formation assays, and wound-healing assays, each biological replicate contained three technical replicates per experimental condition to ensure assay precision. Western blot analyses were performed using three biological replicates, and densitometric quantification reflects the mean values of these independent samples. Technical replicates were averaged before statistical testing so that biological variability served as the basis of statistical inference.

### 2.12. Statistical Analysis

Statistical analyses were performed using the rstatix package in the R programming language [25]. Pairwise comparisons between individual treatment groups and the corresponding control were evaluated using two-tailed unpaired *t*-tests, which were applied only when one experimental condition was compared directly with a single reference group (e.g., colony formation, migration assays, and most Western blot analyses). For experiments involving three or more treatment groups, such as the scratch-wound quantification and collagen deposition assays, data were analyzed using one-way ANOVA followed by Tukey’s post hoc test to correct for multiple comparisons. The replicate strategy followed the standardized framework described in the Reproducibility and Replicates section. Statistical significance was defined as *p* < 0.05. All quantitative data are presented as mean ± standard error (SE) from three independent biological replicates.

## 3. Results

### 3.1. LINCS-Based Target Identification

To identify potential therapeutic candidates for RPF, we employed a transcriptome-guided drug repurposing approach using REMEDY, a LINCS-based computational platform. DEGs were retrieved from a validated radiation-induced lung injury (RILI) mouse model [21], yielding 1023 DEGs at the inflammatory stage and 1423 DEGs at the fibrotic stage. These DEGs served as disease-specific molecular signatures for connectivity analysis.

**Figure 1:**
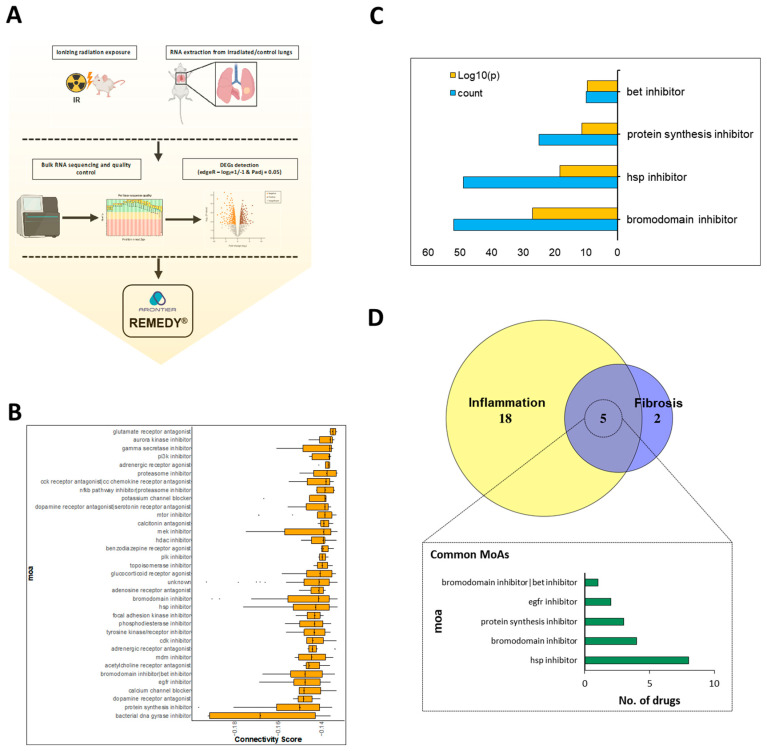
LINCS−driven molecular signature analysis based on target identification. (**A**) Schematic representation of DEGs collection derived from irradiated mice. (**B**) Connectivity score of fibrosis stage signature showing the protein synthesis inhibitor at the top reversed signature. (**C**) MoA-wise analysis comprising count (blue bar) and *p* value (orang bar). (**D**) Venn diagram showing the intersected MoAs of the two groups.

Using REMEDY, reverse connectivity patterns were computed across LINCS L1000 perturbational profiles and ranked according to their negative connectivity scores (Figure 1A). The top signature-reversing compounds for the fibrotic phase are depicted in Figure 1B. Additional analyses supporting the LINCS connectivity results—including extended compound rankings and MoA-level enrichment summaries—are provided in the supplementary figures for clarity and completeness. Specifically, the top reversing compounds for the inflammatory phase are presented in Supplementary Figure S1A, and the corresponding MoA enrichment profiles are shown in Supplementary Figure S1B,C. A Venn diagram comparison revealed the Hsp inhibitors, bromodomain inhibitors, protein synthesis inhibitors, and EGFR inhibitors were commonly identified across datasets (Figure 1D), all of which have been implicated in mitigating radiation-induced effects [26,27,28,29]. These findings prompted our focused investigation of protein synthesis inhibitors as candidate anti-fibrotic agents.

To provide quantitative support for the prioritization of HHT, we calculated signature-level metrics across all radiation-induced DEG profiles (65Gy_2W, 65Gy_6W, 75Gy_2W, 75Gy_6W). HHT consistently demonstrated strong negative connectivity, with z-scores ranging from −0.26 to −0.18, in at least two of these conditions, indicating reversal of RPF-associated transcriptional patterns. FDR-adjusted *p*-values from Fisher’s exact test were below 0.05 for all signatures, and frequency analysis showed that HHT repeatedly appeared within the top 1000 most strongly reverse-connected LINCS perturbagens. These quantitative metrics, summarized in Supplementary Tables S1 and S2, clarify the rationale for prioritizing HHT among protein synthesis inhibitors and support its selection for experimental validation.

To ensure that the connectivity-based identification of HHT was statistically robust, we further evaluated reproducibility across signatures and the stability of our enrichment results. Cross-signature reproducibility analysis showed that the direction and magnitude of HHT’s connectivity scores were preserved at least in two of these DEG signatures. In addition, MoA-level enrichment stability analysis demonstrated that protein synthesis inhibitors remained consistently enriched across all radiation dose-phase combinations. These robustness assessments collectively validate the reproducibility of the computational predictions supporting HHT.

### 3.2. Establishment and Characterization of In Vitro Fibrotic Model

**Figure 2:**
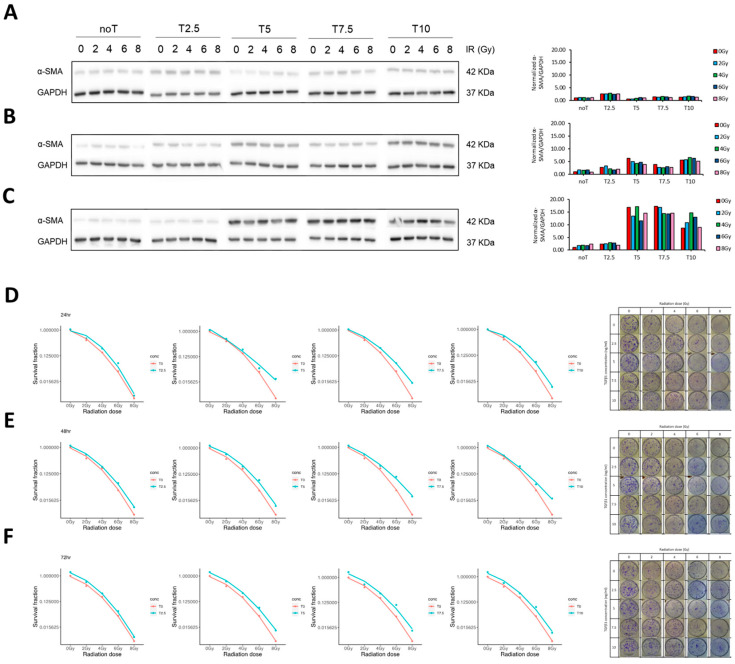
Establishment of in vitro fibrotic model. (**A**–**C**) Western blot showing expression of fibrosis-marker protein, α-SMA at different ionizing radiation (IR) doses, different TGF-β1 concentrations, at 24 h, 48 h, and 72 h. (**D**–**F**) Clonogenic ability of MRC-5 cell using CFA after being treated with different IR doses followed by addition of different concentration of TGF-β1 for 24 h, 48 h, and 72 h, respectively.

To model RPF in vitro, MRC-5 human lung fibroblasts were exposed to IR and stimulated with TGF-β1 to mimic the fibrogenic microenvironment. We systematically optimized IR dose and TGF-β1 concentration to induce myofibroblast differentiation, as indicated by α-SMA expression. Western blot analysis showed peak α-SMA levels at TGF-β1 concentrations of 5 and 7.5 ng/mL after 72 h, while 10 ng/mL induced a slight decline, particularly under high IR conditions (Figure 2A–C).

To identify the optimal conditions for establishing a robust in vitro fibrotic model, we first assessed how IR and TGF-β1 influenced fibroblast growth dymamics. This step was critical to ensure that the chosen experimental parameters would induce fibrogenic activation without significantly compromising cell viability or proliferation capacity. Short-term cell viability was evaluated using the WST-1 assay. The results indicated no significant reduction in cell metabolic activity, even at the highest tested IR doses, suggesting that radiation alone did not acutely impair cellular viability within the 72 h observation window (Supplementary Figure S2). To assess long-term proliferative potential, we performed CFA across a range of IR doses and TGF-β1 concentrations. Treatment with 2 ng/mL TGF-β1 had minimal impact on colony formation. However, higher concentrations (≥5 ng/mL) progressively impaired clonogenic growth, particularly when combined with a high radiation dose (10 Gy), regardless of incubation time. Similarly, radiation doses of 2 and 4 Gy resulted in negligible effects on colony formation, whereas doses ≥6 Gy led to a substantial reduction in colony-forming ability (Figure 2D–F). Based on these findings, we selected a combination of 4 Gy IR and 5 ng/mL TGF-β1 with a 72 h exposure duration as the optimal condition for inducing fibroblast activation while preserving sufficient cell viability for downstream assays.

**Figure 3:**
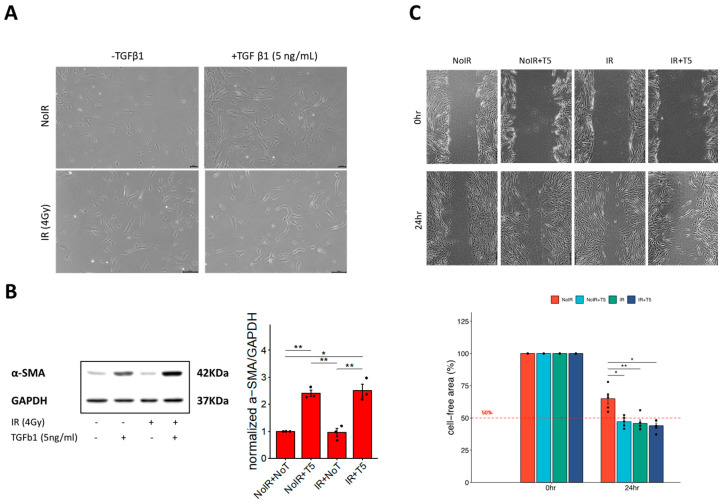
Characterization of in vitro fibrotic model. (**A**) Microscopic observation of MRC-5 cells treated with IR (4 Gy), TGF-β1 (5 ng/mL) only, and IR+TGF-β1. (**B**) Western blot showing expression of the fibrosis-marker protein, α-SMA in the presence of IR, TGF β1, or both treatments. (**C**) Upper panel is a representative scratch-wound images of MRC-5 fibroblasts under control, IR alone, and IR + TGF-β1. IR or TGF-β1 markedly enhanced migratory activity. Lower panel is a quantitative wound-closure analysis showing the percentage of gap closure at 24 h. Red dash refers to threshold for evaluating the migratory trait of activated fibroblast. Data represent mean ± SE from three independent biological replicates, presented as dots, each with three technical replicates. Statistical significance was assessed using one-way ANOVA (*, *p* < 0.05; **, *p* < 0.01).

Following model establishment, we characterized the functional and phenotypic responses of fibroblasts, focusing on cell morphology, differentiation status, and migratory capacity. Microscopic examination revealed that TGF-β1 treatment elicited marked morphological changes consistent with myofibroblast differentiation, regardless of IR exposure (Figure 3A). This was further supported by an increase in α-SMA expression, as confirmed by Western blot analysis (Figure 3B). Interestingly, while α-SMA levels were modestly elevated in cells treated with TGF-β1 alone, the combination of IR and TGF-β1 produced a comparable or slightly augmented effect. One of the most notable changes was in cell size. Untreated fibroblasts typically exhibited diameters of 20–30 µm. In contrast, IR alone induced substantial cell enlargement (150–200 µm), which was further accentuated when combined with TGF-β1 stimulation, suggesting that IR primarily drives cellular hypertrophy.

To assess the migratory behavior of activated fibroblasts, a scratch wound assay was conducted. Both IR and TGF-β1 independently enhanced cell motility, with the most pronounced wound closure observed under combined treatment conditions (Figure 3C). Although a minor, non-significant difference in migration was observed between cells treated with TGF-β1 alone versus those receiving IR plus TGF-β1, the trend suggested a potential additive or synergistic effect.

Collectively, these results demonstrate that IR predominantly contributes to cell size expansion and modestly enhances migration, while TGF-β1 serves as the primary driver of fibroblast differentiation. The combination of both stimuli synergistically amplifies the pro-fibrotic phenotype, validating the suitability of this model for subsequent anti-fibrotic intervention studies.

### 3.3. Protein Synthesis Inhibitor Candidate Inhibited the Proliferation of Activated Fibroblast

**Figure 4:**
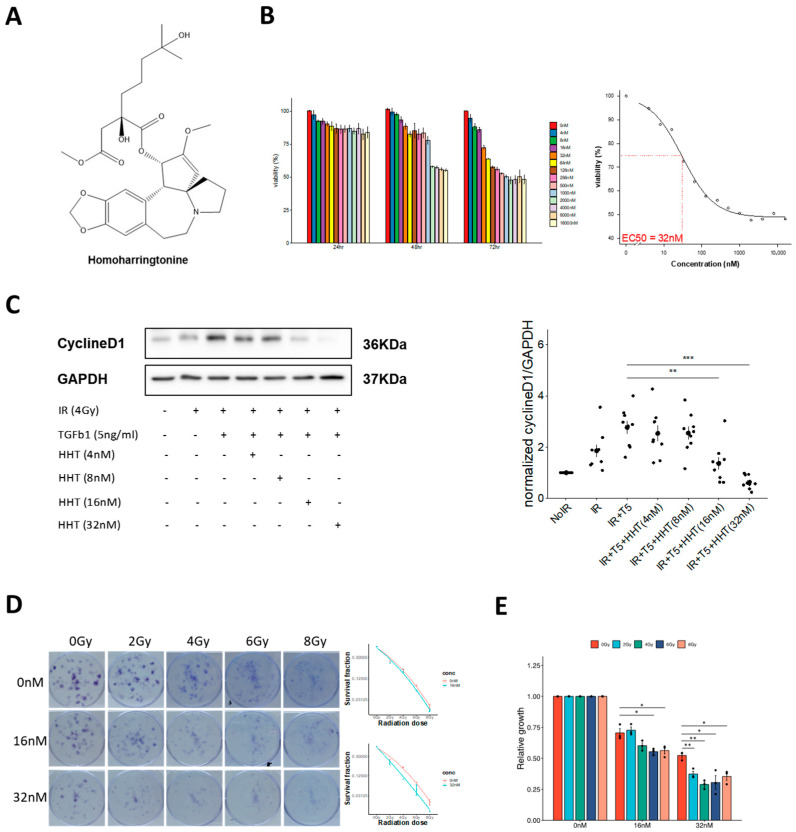
HHT reduces cell viability and clonogenic potential in IR-activated fibroblasts. (**A**) Chemical structure of HHT. (**B**) WST-1 cell viability assay demonstrating dose-dependent reduction in proliferative activity in activated fibroblasts following HHT treatment. (**C**) Western blot showing expression of proliferation biomarker protein cyclin D1 increased following combined application of IR + TGF-β1 and decreased following HHT treatment. (**D**) Left, a representative colony formation images showing that IR + TGF-β1 markedly enhanced clonogenic survival, whereas HHT reduced colony number and size. Right, quantitative clonogenic analysis (colony-forming units) confirming a concentration-dependent decrease in clonogenic capacity with HHT treatment. (**E**) Evaluation of irradiated cell growth relative to control showing irradiated cells more sensitive to HHT inhibition than non-irradiated cells. Data represent mean ± SE from three biological replicates with triplicate technical replicates per condition. Statistical significance was evaluated using two-tailed unpaired *t*-test (*, *p* < 0.05; **, *p* < 0.01; ***, *p* < 0.001).

From our database, three protein synthesis inhibitors, brefeldin-A, emetine, and HHT, were identified as potential candidates. HHT, the only FDA-approved drug among them, was selected for further investigation due to its established clinical use and safety profile (Figure 4A).

Accurate dosage determination was critical to evaluate the biological activity of HHT. Various concentrations were initially tested using the WST-1 cytotoxicity assay, with toxicity assessed at 24, 48, and 72 h. Results indicated a consistent decrease in variability, dropping below 50% at the 72 h mark. The EC50 value was determined to be 32 nm (Figure 4B), and subsequent evaluation were conducted at concentrations ranging from 4 to 32 nm.

To assess HHT’s anti-proliferative effects, potential of the expression of cyclin D1, a proliferation-specific marker, was monitored. Initially, an increase in expression was observed when fibroblasts were exposed to IR alone or in combination with TGF-β1, underscoring its significance in kickstarting the activation-induced proliferation process. Upon the addition of HHT at concentrations of 4 and 8 nm, a modest reduction in cyclin D1 expression was noted, whereas concentrations of 16 and 32 nm notably suppressed its expression (Figure 4C).

These findings were corroborated by a CFA, which provided long-term functional validation of HHT’s anti-proliferative effects. At 32 nm, HHT markedly suppressed colony-forming ability, with a more pronounced inhibitory effect in irradiated (activated) fibroblasts compared to non-irradiated (resident) cells (Figure 4D,E). This differential sensitivity suggests that HHT preferentially targets pathologically activated fibroblasts while sparing quiescent populations.

Together, these results establish HHT as a potent suppressor of fibroblast proliferation under fibrotic conditions and support its potential as a therapeutic agent for mitigating RPF.

### 3.4. HHT Inhibited Fibroblast Differentiation by Impacting the Rock/RhoA Signaling Pathway

**Figure 5:**
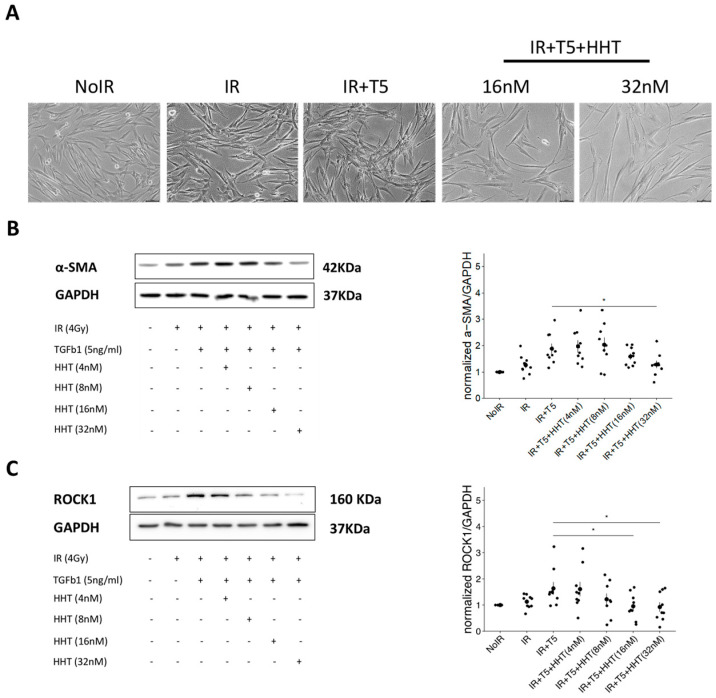
HHT inhibited differentiation of IR-activated fibroblasts through targeting ROCK/RhoA signaling pathway. (**A**) Representative phase-contrast microscopic images of MRC-5 fibroblasts under control, IR alone, IR + TGF-β1, and IR + TGF-β1 with HHT treatment conditions. IR + TGF-β1 induced pronounced morphological changes, including increased cell size, cytoskeletal thickening, and pericellular membrane formation, all of which were attenuated by HHT in a dose-dependent manner. (**B**) Western blot analysis of α-SMA expression demonstrating significant suppression of myofibroblast differentiation upon HHT treatment. (**C**) Western blot analysis of ROCK1 expression showing that HHT reduced ROCK1 levels toward those of non-activated control fibroblasts. Densitometric quantifications for (**B**,**C**) are presented as mean ± SE from three biological replicates, represented by dotes in each group. Statistical significance was assessed using two-tailed unpaired *t*-test (*, *p* < 0.05).

Microscopic analyses were conducted to evaluate the effects of HHT on fibroblast differentiation and cytoskeletal stiffness under fibrotic conditions. Exposure to IR alone induced partial differentiation and pericellular membrane formation, whereas subsequent treatment with TGF-β1 promoted full myofibroblast transformation, characterized by enhanced membrane development and increased cellular stiffness. Notably, HHT treatment effectively reversed these phenotypic changes, reducing both pericellular membrane formation and stiffness in a dose-dependent manner (Figure 5A).

To confirm the inhibitory effect of HHT on myofibroblast differentiation, the expression of α-SMA, a key marker of myofibroblastic transition, was assessed. Western blot analysis revealed a concentration-dependent reduction in α-SMA expression, indicating that HHT significantly attenuates fibroblast differentiation into the myofibroblast phenotype (Figure 5B).

We next examined the expression of ROCK1, a critical downstream effector in the RhoA/ROCK signaling pathway involved in stress fiber formation and contractility. HHT treatment markedly suppressed ROCK1 expression in a dose-dependent fashion, with the highest concentration (32 nm) restoring ROCK1 levels to near those observed in non-irradiated control cells (Figure 5C). These findings suggest that HHT effectively inhibits fibroblast differentiation and stiffness by targeting the RhoA/ROCK pathway, supporting its potential utility as a therapeutic agent to mitigate radiation-induced fibrogenesis.

### 3.5. HHT Suppressed Extracellular Matrix Production via Influencing Wnt/β-Catenin Pathway

**Figure 6:**
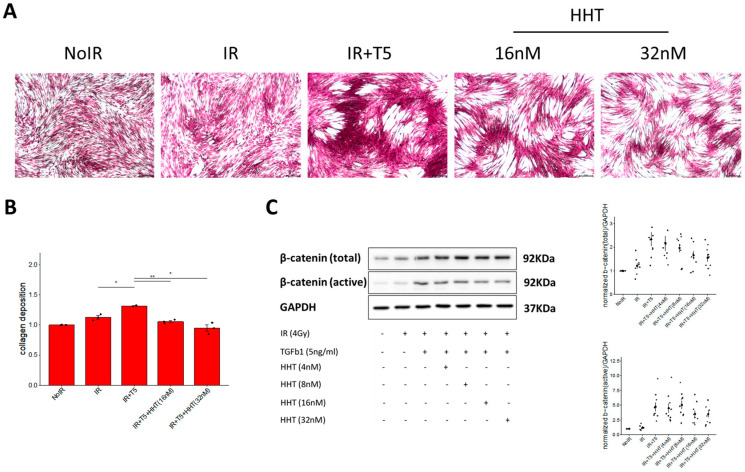
HHT suppressed collagen production by IR-activated fibroblasts through targeting Wnt/β-catenin signaling pathway. (**A**) Representative microscopic images of collagen deposition staining of MRC-5 fibroblasts under control, IR alone, IR + TGF-β1, and IR + TGF-β1 with HHT treatment conditions. (**B**) Quantitative analysis of collagen deposition assessed by Direct Red 80 staining. HHT significantly reduced IR + TGF-β1–induced collagen accumulation in a dose-dependent manner. Data are presented as mean ± SE from three biological replicates. (**C**) Western blot analysis of total and active β-catenin expression in MRC-5 cells under the same conditions. HHT progressively attenuated β-catenin upregulation induced by IR + TGF-β1. Corresponding densitometric quantification is shown. Statistical significance was evaluated using two-tailed unpaired *t*-test (*, *p* < 0.05; **, *p* < 0.01).

Precise regulation of ECM components, such as collagen, is essential for maintaining tissue homeostasis and supporting wound healing. Aberrant ECM accumulation is a hallmark of fibrotic diseases, including RPF. To investigate whether HHT can suppress ECM production, we conducted a collagen staining assay using Direct Red 80, in which staining intensity correlates with the amount of collagen deposition. Moderate collagen staining was observed in cells treated with IR alone, whereas cells co-treated with IR and TGF-β1 exhibited markedly enhanced staining, reflecting increased collagen synthesis. The addition of HHT at 16 nm and 32 nm significantly attenuated collagen deposition in a dose-dependent manner. Quantitative image analysis revealed that IR alone increased collagen production by approximately 10%, while IR + TGF-β1 led to a 31% increase. HHT treatment substantially reversed this effect, restoring collagen levels to those comparable to untreated control cells (Figure 6B). To further validate the anti-fibrotic action of HHT, we analyzed the expression of β-catenin, a key transcriptional effector in the canonical Wnt signaling pathway, which is known to regulate ECM gene expression. Western blot results demonstrated that IR + TGF-β1 markedly increased β-catenin levels, whereas HHT administration progressively reduced its expression in a concentration-dependent manner (Figure 6C).

**Figure 7:**
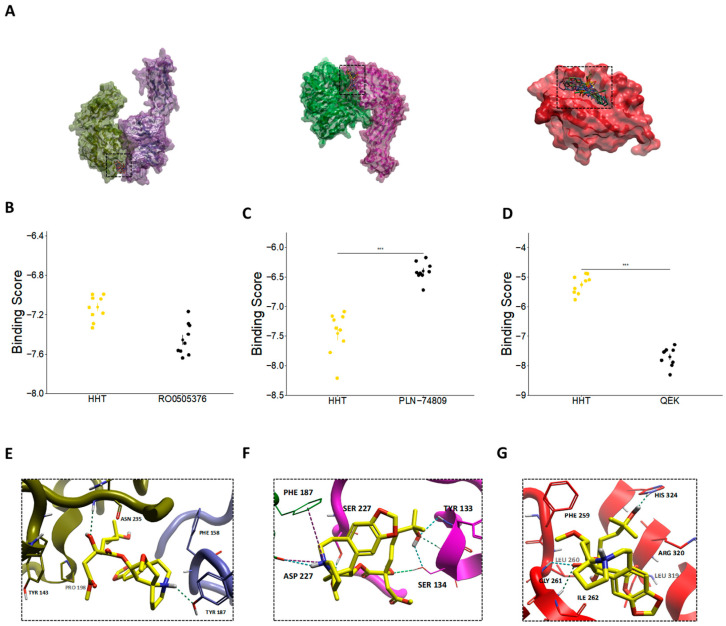
Molecular docking analysis of HHT with integrin (α4β7, α5β1) and Frizzled (FzD4) receptors. (**A**) Global overview of the top 10 predicted docking poses of HHT bound to α4β7, α5β1, and FzD4, key receptors associated with RhoA/ROCK and Wnt/β-catenin signaling pathways. (**B**–**D**) Predicted binding free energies (ΔG) of HHT (yellow-colored) compared with the respective reference ligands (black-colored)—RO0505376 for α4β7, PLN-74809 for α5β1, and QEK for FzD4. ΔΔG values (ΔG_HHT–ΔG_reference) are indicated to enable direct comparison of binding favorability. (**E**–**G**) Local interaction maps illustrating hydrogen-bonding and hydrophobic contacts between HHT and key amino acid residues within the binding pockets of (**E**) α4β7, (**F**) α5β1, and (**G**) FzD4. Structures and interactions were visualized using Flare Viewer v10. Statistical significance was assessed using two-tailed unpaired *t*-test (***, *p* < 0.001).

Collectively, these findings demonstrate that HHT suppresses ECM production in activated lung fibroblasts by modulating the Wnt/β-catenin signaling axis, highlighting its promise as a therapeutic candidate for attenuating fibrotic remodeling in RPF. To complement these molecular and phenotypic findings, we performed a comparative docking analysis to evaluate the plausibility of direct receptor engagement by HHT. Predicted binding free energies (ΔG) demonstrated that HHT binds favorably to all three receptors examined—α4β7, α5β1, and FzD4. The calculated ΔΔG values (ΔG_HHT–ΔG_reference) showed that HHT displayed comparable or more favorable affinity than the known ligands, specially for intgerins receptors. Local interaction maps revealed that HHT forms stable hydrogen-bonding and hydrophobic contacts with key residues within each receptor pocket, supporting the feasibility of these interactions (Figure 7B–D).

### 3.6. HHT May Directly Impact the Signaling Pathways via Non-Protein Synthesis Inhibition Mechanism

To explore whether HHT directly interferes with pro-fibrotic signaling pathways beyond its canonical role as a protein synthesis inhibitor, we performed molecular docking simulations targeting key receptors involved in the ROCK/RhoA and Wnt/β-catenin pathways. Specifically, we investigated HHT’s binding affinity to integrins α4β7 and α5β1, which regulate ROCK-mediated cytoskeletal remodeling and cell contractility through their interactions with ECM components such as fibronectin. Established inhibitors—RO0505376 for α5β1—exert their effects by binding to well-characterized sites, including the metal ion-dependent adhesion site (MIDAS) and the RGD-binding pocket, respectively [30].

In parallel, we assessed FzD receptors, which are key transmembrane proteins in the Wnt/β-catenin signaling cascade. FzD receptors promote β-catenin nuclear translocation by interacting with Wnt ligands via their Wnt-binding domain and the cytoplasmic Disheveled (Dvl)-binding domain. Small-molecule inhibitors such as compound 18 (QEK) disrupt Wnt signaling by targeting the Dvl-binding interface, thereby suppressing β-catenin activation [31].

Docking simulations revealed that HHT binds favorably within the ligand-binding pockets of both α4β7 and α5β1 integrins, yielding predicted free energy scores that were comparable to or better than those of their respective reference inhibitors (Figure 7A,B). While HHT exhibited relatively lower predicted binding affinity for the Dvl domain of FzD—requiring more energy for binding than the known inhibitor PLN-74809 (Figure 7D)—it demonstrated improved docking performance at the cysteine-rich domain (CRD) of FzD, particularly in the homodimeric configuration (Supplementary Figure S3).

Residue-level interaction analyses further substantiated these findings. In the α4β7 complex, HHT was predicted to interact with TYR187 of the α4 subunit and with TYR143 and ASN235 of the β7 subunit (Figure 7E). In α5β1, HHT engaged the RGD-binding interface through contacts with TYR133 of the α5 subunit and ASP227 of the β1 subunit (Figure 7F). Although its binding affinity for FzD was comparatively weaker, HHT formed notable interactions with ILE262, GLY261, PHE259, HIS324, and ARG320—residues implicated in ligand recognition and conformational switching (Figure 7G).

These results suggest that HHT may function as a competitive ligand capable of directly engaging integrin and Frizzled receptors, thereby modulating the ROCK/RhoA and Wnt/β-catenin signaling pathways. This potential for direct receptor-level inhibition complements its translational inhibition properties and provides additional mechanistic support for HHT as a candidate therapeutic for fibrotic disorders such as RPF. While these docking simulations provide structural hypotheses suggesting that HHT may directly engage integrin and Frizzled receptors, it is important to emphasize that computational docking predicts binding poses and approximate relative affinities based on simplified scoring functions. These methods do not account for full protein flexibility, solvent dynamics, or conformational changes. Therefore, the docking findings in Section 3.6 should be interpreted as hypothesis-generating rather than confirmatory, and experimental biochemical validation (e.g., binding assays, mutagenesis mapping, SAR studies) will be required to verify these predicted interactions.

## 4. Discussion

**Figure 8:**
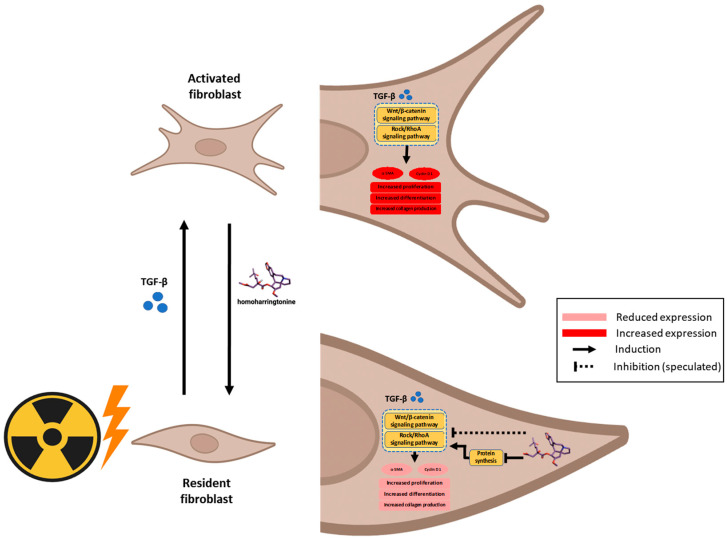
Illustrative scheme describing the proposed mechanism of action of HHT driving the anti-fibrotic effect on activated fibroblasts via impacting Wnt/β-catenin and Rock/RhoA signaling pathways.

Genome-wide association studies (GWAS) have identified numerous genetic loci associated with disease susceptibility. However, listing these loci alone often fails to establish causality or provide mechanistic insight. To bridge this gap, perturbation-based approaches—where cellular systems are modulated and responses observed—are crucial for elucidating disease mechanisms and identifying therapeutic targets. The CMap, which links genes, drugs, and diseases via shared gene expression signatures, offers a powerful platform for understanding the mechanisms of small molecules. In this study, we employed REMEDY, a computational drug repurposing platform built on the LINCS dataset, to identify candidate therapeutics for RPF. Through this approach, we identified protein synthesis inhibitors—particularly HHT—as top-ranked candidates. Subsequent in vitro validation using irradiated, TGF-β1-activated fibroblasts confirmed the anti-fibrotic effects of HHT (Figure 8).

While HHT’s anti-fibrotic activity has been described in arthrofibrosis, hepatic fibrosis, and cardiac fibrosis, these conditions do not share the same transcriptomic architecture as radiation-induced injury. Fibrosis biology exhibits a high degree of pathway convergence, particularly involving ROCK/RhoA and Wnt/β-catenin signaling. Therefore, overlap in mechanistic pathways is biologically expected and does not diminish the originality of the present work. The novelty of this study lies in demonstrating that RPF-specific molecular signatures can computationally predict HHT as a reverse-signature therapeutic candidate and in validating its effects within a radiation-dependent fibroblast activation model, a context that has never previously been explored.

Recent tools such as L1000 Fireworks Display (L1000FWD), the Drug Gene Interaction Database (DGIdb), and CMap have been increasingly utilized to predict drug–disease interactions by comparing disease-associated DEGs with drug-induced gene signatures [14]. This strategy has demonstrated success across various pathologies, including cancer [16,17], muscle atrophy [18], acute myelogenous leukemia [19], and Parkinson’s disease [20]. To our knowledge, the present study represents the first application of the LINCS/CMap platform to identify therapeutic candidates for RPF.

Establishing a relevant in vitro model that mimics the RPF microenvironment was a foundational step in this study. Previous studies typically relied on TGF-β1 alone to induce fibroblast activation and differentiation into myofibroblasts. However, this does not fully recapitulate the pathological conditions of radiation-induced lung injury (RILI). Here, we combined IR with TGF-β1 to establish a more physiologically relevant fibrotic model. Our results demonstrated that α-SMA—a hallmark of myofibroblast differentiation—was robustly upregulated following treatment with 5 ng/mL TGF-β1 at 72 h, even in the absence of IR. This finding partially contrasts prior reports where α-SMA induction was IR-dependent and further enhanced by TGF-β1 [32]. Another important methodological consideration in establishing our in vitro RPF model was the selection of the radiation dose. We systematically evaluated a range of doses (0–8 Gy) to determine the optimal balance between fibroblast activation and cell viability. Our data showed that 4 Gy produced the most robust induction of α-SMA and stress-fiber remodeling, whereas lower doses such as 2 Gy induced only minimal activation despite better overall survival. In contrast, higher doses (6–8 Gy) markedly reduced clonogenic capacity and increased apoptosis, thereby limiting their suitability for downstream functional assays. Thus, 4 Gy represented an optimal “activation-without-cytotoxicity” window—sufficient to synergize with TGF-β1 and reproduce a reproducible fibrotic phenotype while maintaining viable cell populations for mechanistic and phenotypic analyses. This rationale supports the use of 4 Gy as the irradiation condition for the fibrotic model employed in this study.

It is important to note that previous studies employed higher IR doses (e.g., 6 Gy), which may have contributed to greater α-SMA expression. In our model, we used a 4 Gy dose to minimize radiation-induced apoptosis, which was observed to increase sharply at higher doses. We hypothesize that α-SMA induction in our model may be driven largely by TGF-β1, with IR contributing more to cell enlargement and migration than differentiation.

This also aligns with prior observations showing that low IR doses (e.g., 3 Gy) combined with TGF-β1 may not significantly increase α-SMA expression [33]. Additionally, our model demonstrated IR- and TGF-β1-dependent enhancement of fibroblast migration, further validating its fidelity. The concurrence between our data and previous findings confirms that this two-hit model reliably reproduces key fibrogenic features and is suitable for testing anti-fibrotic agents.

HHT is a well-characterized plant alkaloid and FDA-approved drug for the treatment of chronic myeloid leukemia. Recently, its therapeutic potential has been explored in various fibrotic conditions, including arthrofibrosis [34], epidural fibrosis [35], liver fibrosis [17], and cardiac fibrosis [36]. However, our study is the first to propose HHT as a candidate for treating RPF, specifically during the fibrotic phase. Prior research has reported effective anti-fibrotic activity at a concentration of ~34 nm [36], which is consistent with the dose range employed in our experiments. Notably, HHT exhibited stronger anti-proliferative effects in irradiated (activated) fibroblasts compared to non-irradiated cells, suggesting preferential targeting of pathological phenotypes with reduced toxicity to normal tissue. Several molecular parallels exist between RPF and idiopathic pulmonary fibrosis (IPF), including epithelial–mesenchymal transition (EMT), excessive ECM deposition, and myofibroblast-driven lung tissue remodeling. The RhoA/ROCK pathway plays a central role in fibrogenesis by promoting fibroblast contractility, α-SMA expression, and collagen production. Dual inhibition of ROCK1 and ROCK2 has been shown to suppress COL1A1 and α-SMA expression and disrupt stress fiber formation in both animal models and MRC-5 cell lines [37,38]. Likewise, the canonical Wnt/β-catenin pathway is implicated in fibroblast activation and ECM production. Inhibition of β-catenin nuclear translocation—e.g., by bufotalin—has been shown to attenuate lung fibrosis in both bleomycin- and radiation-induced models [39]. Given that pro-fibrotic remodeling is mediated by a limited set of conserved molecular pathways, most anti-fibrotic agents—including HHT—are naturally expected to converge on these canonical nodes. Accordingly, our goal was not to propose a novel molecular target for HHT, but to establish its relevance to radiation-triggered fibroblast activation using RPF-specific transcriptomic data. This distinction is essential, as the radiation-induced fibrotic environment differs markedly from idiopathic or post-surgical fibrosis, both in cytokine composition and in gene-expression patterning. By repositioning HHT to this radiation-specific disease context, our study fills a previously unaddressed therapeutic gap.

A limitation of the present study is the absence of established anti-fibrotic agents such as pirfenidone or nintedanib as positive controls. Although these agents are commonly used in preclinical fibrosis studies, including radiation-associated models, their effects in fibroblast-based assays are generally modest and often require prolonged exposure or more complex systems (e.g., co-culture or ECM-enriched conditions) to yield quantifiable changes. Because our primary aim was to conduct initial mechanistic and phenotypic validation of a transcriptome-identified hit (HHT) using a simplified IR/TGF-β1 activation model, we compared HHT only with the IR-treated negative control to isolate its direct influence on fibroblast activation without confounding pharmacologic background. Nonetheless, future work incorporating pirfenidone and nintedanib using extended culture periods and 3D/ECM-enhanced models is underway and will help benchmark the comparative potency and mechanistic distinctiveness of HHT.

An important consideration when repurposing HHT for a non-malignant indication such as RPF is its well-known myelosuppressive toxicity profile. In oncology, the dose-limiting toxicity of HHT is systemic myelosuppression, largely driven by its inhibition of global protein synthesis in rapidly proliferating hematopoietic cells. However, the concentrations applied in our in vitro experiments (10–50 nm) are substantially lower than those used in leukemia treatment, suggesting the possibility of a wider therapeutic window for applications that employ localized, low-dose, or short-exposure strategies. Moreover, several drug-delivery approaches—including localized pulmonary delivery, nanoformulation, liposomal encapsulation, and inhalation-based aerosolization—have been investigated in prior preclinical studies of other repurposed agents to limit systemic exposure while maximizing target-organ deposition. Although such delivery strategies were beyond the scope of the present study, they may theoretically reduce bone-marrow exposure and warrant systematic evaluation in future preclinical toxicology studies. Taken together, these considerations underscore the importance of carefully assessing HHT’s safety profile during its translational development while also highlighting feasible avenues to mitigate systemic toxicity as the compound advances toward in vivo investigation.

Despite the strengths of our computational–experimental workflow, several limitations should be acknowledged. First, the present study lacks in vivo validation, and future work using radiation-induced lung fibrosis models will be required to determine whether the transcriptome-guided effects of HHT translate into organism-level mitigation of fibrotic remodeling. Second, although we discuss potential strategies to limit systemic exposure, concerns regarding off-target or non-pulmonary toxicity remain and must be rigorously addressed in dedicated preclinical toxicology studies. Third, our computational predictions were derived exclusively from the LINCS L1000 dataset, which, despite its scale, captures only a subset of drug-induced transcriptional states. Additional cross-platform validation—incorporating alternative perturbational resources or full-length RNA-seq signatures—will therefore be important to strengthen the generalizability of our drug-repurposing results.

In addition, the docking analyses should be viewed as predictive and hypothesis-generating rather than definitive biochemical evidence of receptor–ligand binding. AutoDock Vina scoring functions approximate relative free energies and do not capture full receptor flexibility, solvent dynamics, or induced-fit conformational changes. Consequently, the predicted interactions between HHT and integrin or Frizzled receptors should be interpreted cautiously, and future biochemical binding and SAR studies will be required to validate these proposed interactions.

Collectively, these elements highlight that the originality of this study resides not in discovering a new biochemical action of HHT, but in demonstrating that a LINCS-based signature reversion strategy tailored to RPF can identify a viable therapeutic compound. This represents a conceptual advance in how radiation-related fibrotic disorders may be approached, enabling therapeutic discovery that is guided by disease-specific transcriptomic signatures rather than generalized fibrosis pathways.

Our data show that HHT significantly suppresses α-SMA, ROCK1, and β-catenin expression, consistent with inhibition of both the RhoA/ROCK and Wnt/β-catenin pathways. These results provide mechanistic support for HHT’s anti-fibrotic action and highlight its potential to interrupt multiple pro-fibrotic signaling cascades. Importantly, the anti-fibrotic effects observed with HHT cannot be explained solely by non-specific cytotoxicity arising from its canonical role as a protein synthesis inhibitor. First, the concentrations used in our in vitro assays (10–50 nm) fall within a range reported to exert minimal global cytotoxicity in fibroblast systems, yet these doses led to substantial reductions in α-SMA, ROCK1, and β-catenin expression. Second, inhibition of fibroblast migration occurred at concentrations that did not significantly affect cell viability, indicating a functional anti-fibrotic response independent of cell death. Third, irradiated fibroblasts displayed heightened sensitivity to HHT compared with non-irradiated cells, suggesting preferential targeting of activated or pathological phenotypes rather than generalized toxicity. Finally, molecular docking revealed strong binding affinities of HHT to α5β1, α4β7, and FzD receptors, supporting a mechanism that extends beyond translation inhibition alone. Together, these findings indicate that HHT mediates pathway-level modulation and phenotypic reprogramming rather than exerting merely translation-related cytotoxicity. Integrins, particularly α4- and α5-containing subtypes (e.g., α4β7, α5β1), are key regulators of fibrosis through their ability to activate RhoA/ROCK signaling and promote cytoskeletal reorganization and matrix stiffening [40,41,42]. Prior studies have shown that HHT inhibits α5β1-mediated signaling in bladder cancer cells, either through direct receptor interaction or via suppression of protein translation [43]. In our molecular docking simulations, HHT demonstrated strong binding affinities to α5β1, α4β7, and FzD receptors, further supporting its ability to modulate fibrosis-relevant pathways beyond translation inhibition.

## 5. Conclusions

In this study, we employed a LINCS-based computational drug repurposing strategy using the REMEDY platform to identify novel therapeutic candidates for RPF. Among the top hits, HHT—an FDA-approved protein synthesis inhibitor—was prioritized based on its high reverse connectivity score and clinical availability.

Transcriptomic analysis of a RILI model revealed key fibrogenic pathways and molecular targets that were effectively modulated by HHT. Subsequent in vitro validation demonstrated that HHT significantly inhibited fibroblast proliferation, differentiation, ECM production, and cellular stiffness. Mechanistically, HHT suppressed critical fibrosis-associated pathways, including RhoA/ROCK and Wnt/β-catenin signaling. In silico molecular docking further supported the potential for direct interaction of HHT with integrin and Frizzled receptors, providing insight into its multi-modal mechanism of action.

Collectively, these findings position HHT as a promising anti-fibrotic agent for RPF. This work lays the foundation for future preclinical studies and supports the potential translation of HHT into a viable therapeutic option for managing radiation-induced fibrotic lung disease. However, considering known myelosuppresive effect of HHT, further in vivo validation, pharmacological optimization, and proper drug administration will be essential to fully establish its safety, efficacy, and clinical applicability.

## Data Availability

The raw data associated with this study will be provided upon request.

## Supplementary Materials

The following supporting information can be downloaded at: https://www.mdpi.com/article/10.3390/pharmaceutics17121626/s1, Supplementary Figure S1: Molecular signature of inflammation process; Supplementary Figure S2: Viability assay of MRC-5 using WST-1 assay; Supplementary Figure S3: Molecular docking analysis of HHT against homodimeric frizzled receptor. Supplementary Table S1: list of ranked perturbagens using DEGs of early stage 65 Gy irradiated mice; Supplementary Table S2: list of ranked perturbagens using DEGs of early stage 75 Gy irradiated mice.

## Abbreviations

The following abbreviations are used in this manuscript:

IR	Ionizing radiation
RPF	Radiation-induced pulmonary fibrosis
RILI	Radiation-induced lung fibrosis
TGF-β1	Transforming growth factor-β1
TNF-α	Tumor necrosis factor-α
IL-1β	Interleukin-1β
IL-6	Interleukin-6
ECM	Extracellular matrix
α-SMA	α-smooth muscle actin
GEO	Gene Expression Omnibus
CMap	Connectivity Map
LINCS	Library of Integrated Network-Based Cellular Signatures
DEGs	Differentially expressed genes
HHT	homoharringtonine
MoA	Mode of Action
KS	Kolmogorov–Smirnov
PDB	Protein Data Bank
EMT	Epithelial–mesenchymal transition
CFA	Colony formation assay
FzD	Frizzled
MIDAS	metal ion-dependent adhesion site
Dvl	Disheveled
DGIdb	Drug Gene Interaction Database
(L1000FWD)	L1000 Fireworks Display

## References

[1] Simone C.B., Wildt B., Haas A.R., Pope G., Rengan R., Hahn S.M. (2013). Stereotactic Body Radiation Therapy for Lung Cancer. Chest.

[2] Mehta V. (2005). Radiation Pneumonitis and Pulmonary Fibrosis in Non-Small-Cell Lung Cancer: Pulmonary Function, Prediction, and Prevention. Int. J. Radiat. Oncol. Biol. Phys..

[3] Arroyo-Hernández M., Maldonado F., Lozano-Ruiz F., Muñoz-Montaño W., Nuñez-Baez M., Arrieta O. (2021). Radiation-Induced Lung Injury: Current Evidence. BMC Pulm. Med..

[4] Hosseini S.A., Zahedipour F., Sathyapalan T., Jamialahmadi T., Sahebkar A. (2021). Pulmonary Fibrosis: Therapeutic and Mechanistic Insights into the Role of Phytochemicals. Biofactors.

[5] Tai Y., Woods E.L., Dally J., Kong D., Steadman R., Moseley R., Midgley A.C. (2021). Myofibroblasts: Function, Formation, and Scope of Molecular Therapies for Skin Fibrosis. Biomolecules.

[6] Kim E.S., Keating G.M. (2015). Pirfenidone: A Review of Its Use in Idiopathic Pulmonary Fibrosis. Drugs.

[7] Cho M.E., Kopp J.B. (2010). Pirfenidone: An Anti-Fibrotic and Cytoprotective Agent as Therapy for Progressive Kidney Disease. Expert. Opin. Investig. Drugs.

[8] Wollin L., Wex E., Pautsch A., Schnapp G., Hostettler K.E., Stowasser S., Kolb M. (2015). Mode of Action of Nintedanib in the Treatment of Idiopathic Pulmonary Fibrosis. Eur. Respir. J..

[9] Wollin L., Distler J.H.W., Redente E.F., Riches D.W.H., Stowasser S., Schlenker-Herceg R., Maher T.M., Kolb M. (2019). Potential of Nintedanib in Treatment of Progressive Fibrosing Interstitial Lung Diseases. Eur. Respir. J..

[10] Scannell J.W., Blanckley A., Boldon H., Warrington B. (2012). Diagnosing the Decline in Pharmaceutical R&D Efficiency. Nat. Rev. Drug Dis..

[11] Ashburn T.T., Thor K.B. (2004). Drug Repositioning: Identifying and Developing New Uses for Existing Drugs. Nat. Rev. Drug Dis..

[12] Liu Z., Fang H., Reagan K., Xu X., Mendrick D.L., Slikker W., Tong W. (2013). In Silico Drug Repositioning—What We Need to Know. Drug Discov. Today.

[13] Li J., Zheng S., Chen B., Butte A.J., Swamidass S.J., Lu Z. (2016). A Survey of Current Trends in Computational Drug Repositioning. Brief. Bioinform..

[14] Lamb J., Crawford E.D., Peck D., Modell J.W., Blat I.C., Wrobel M.J., Lerner J., Brunet J.-P.P., Subramanian A., Ross K.N. (2006). The Connectivity Map: Using Gene-Expression Signatures to Connect Small Molecules, Genes, and Disease. Science.

[15] Subramanian A., Narayan R., Corsello S.M., Peck D.D., Natoli T.E., Lu X., Gould J., Davis J.F., Tubelli A.A., Asiedu J.K. (2017). A Next Generation Connectivity Map: L1000 Platform and the First 1,000,000 Profiles. Cell.

[16] Sirota M., Dudley J.T., Kim J., Chiang A.P., Morgan A.A., Sweet-Cordero A., Sage J., Butte A.J. (2011). Discovery and Preclinical Validation of Drug Indications Using Compendia of Public Gene Expression Data. Sci. Transl. Med..

[17] Yang C., Zhang H., Chen M., Wang S., Qian R., Zhang L., Huang X., Wang J., Liu Z., Qin W. (2022). A Survey of Optimal Strategy for Signature-Based Drug Repositioning and an Application to Liver Cancer. eLife.

[18] Kunkel S.D., Suneja M., Ebert S.M., Bongers K.S., Fox D.K., Malmberg S.E., Alipour F., Shields R.K., Adams C.M. (2011). MRNA Expression Signatures of Human Skeletal Muscle Atrophy Identify a Natural Compound That Increases Muscle Mass. Cell Metab..

[19] Hassane D.C., Guzman M.L., Corbett C., Li X., Abboud R., Young F., Liesveld J.L., Carroll M., Jordan C.T. (2008). Discovery of Agents That Eradicate Leukemia Stem Cells Using an in Silico Screen of Public Gene Expression Data. Blood.

[20] Gao L., Zhao G., Fang J.S., Yuan T.Y., Liu A.L., Du G.H. (2014). Discovery of the Neuroprotective Effects of Alvespimycin by Computational Prioritization of Potential Anti-Parkinson Agents. FEBS J..

[21] Farh M.E.A., Kim H.J., Kim S.Y., Lee J.H., Lee H., Cui R., Han S., Kim D.W., Park S., Lee Y.J. (2024). Transcriptional Changes in Radiation-Induced Lung Injury: A Comparative Analysis of Two Radiation Doses for Preclinical Research. Int. J. Mol. Sci..

[22] Goodsell D.S., Olson A.J. (1990). Automated Docking of Substrates to Proteins by Simulated Annealing. Proteins Struct. Funct. Bioinform..

[23] O’Boyle N.M., Banck M., James C.A., Morley C., Vandermeersch T., Hutchison G.R. (2011). Open Babel: An Open Chemical Toolbox. J. Cheminform..

[24] Trott O., Olson A.J. (2010). AutoDock Vina: Improving the Speed and Accuracy of Docking with a New Scoring Function, Efficient Optimization, and Multithreading. J. Comput. Chem..

[25] Rstatix R Package Citation Profile. https://scholar.rpkg.net/pkg/rstatix.

[26] Wang J., Zhou F., Li Z., Mei H., Wang Y., Ma H., Shi L., Huang A., Zhang T., Lin Z. (2018). Pharmacological Targeting of BET Proteins Attenuates Radiation-Induced Lung Fibrosis. Sci. Rep..

[27] Miyake K., Tani K., Kakiuchi S., Suzuka C., Toyoda Y., Kishi J., Tezuka T., Yuasa S., Hanibuchi M., Aono Y. (2012). Epidermal Growth Factor Receptor-Tyrosine Kinase Inhibitor (Gefitinib) Augments Pneumonitis, but Attenuates Lung Fibrosis in Response to Radiation Injury in Rats. J. Med. Investig..

[28] Valinciute G., Weigel C., Veldwijk M.R., Oakes C.C., Herskind C., Wenz F., Plass C., Schmezer P., Popanda O. (2017). BET-Bromodomain Inhibitors Modulate Epigenetic Patterns at the Diacylglycerol Kinase Alpha Enhancer Associated with Radiation-Induced Fibrosis. Radiother. Oncol..

[29] Li L., Wu D., Deng S., Li J., Zhang F., Zou Y., Zhang T., Xu Y. (2022). NVP-AUY922 Alleviates Radiation-Induced Lung Injury via Inhibition of Autophagy-Dependent Ferroptosis. Cell Death Discov..

[30] Slack R.J., Macdonald S.J.F., Roper J.A., Jenkins R.G., Hatley R.J.D. (2022). Emerging Therapeutic Opportunities for Integrin Inhibitors. Nat. Rev. Drug Discov..

[31] Kamdem N., Roske Y., Kovalskyy D., Platonov M.O., Balinskyi O., Kreuchwig A., Saupe J., Fang L., Diehl A., Schmieder P. (2021). Small-Molecule Inhibitors of the PDZ Domain of Dishevelled Proteins Interrupt Wnt Signalling. Magn. Reson..

[32] Shao L., Zhang Y., Shi W., Ma L., Xu T., Chang P., Dong L. (2021). Mesenchymal Stromal Cells Can Repair Radiation-Induced Pulmonary Fibrosis via a DKK-1-Mediated Wnt/β-Catenin Pathway. Cell Tissue Res..

[33] Kumar D., Yalamanchali S., New J., Parsel S., New N., Holcomb A., Gunewardena S., Tawfik O., Lominska C., Kimler B.F. (2018). Development and Characterization of an in Vitro Model for Radiation-Induced Fibrosis. Radiat. Res..

[34] Sun Y., Dai J., Jiao R., Jiang Q., Wang J. (2021). Homoharringtonine Inhibits Fibroblasts Proliferation, Extracellular Matrix Production and Reduces Surgery-Induced Knee Arthrofibrosis via PI3K/AKT/MTOR Pathway-Mediated Apoptosis. J. Orthop. Surg. Res..

[35] Li X., Wang S., Dai J., Yan L., Zhao S., Wang J., Sun Y. (2017). Homoharringtonine Prevents Surgery-Induced Epidural Fibrosis through Endoplasmic Reticulum Stress Signaling Pathway. Eur. J. Pharmacol..

[36] Kreutzer F.P., Meinecke A., Mitzka S., Hunkler H.J., Hobuß L., Abbas N., Geffers R., Weusthoff J., Xiao K., Jonigk D.D. (2022). Development and Characterization of Anti-Fibrotic Natural Compound Similars with Improved Effectivity. Basic. Res. Cardiol..

[37] Liu L., Song W., Zeng J., Peng Z. (2022). Evaluating a Specific Dual ROCK Inhibitor against Bleomycin-Induced Idiopathic Pulmonary Fibrosis in Rats. ACS Pharmacol. Transl. Sci..

[38] Knipe R.S., Probst C.K., Lagares D., Franklin A., Spinney J.J., Brazee P.L., Grasberger P., Zhang L., Black K.E., Sakai N. (2018). The Rho Kinase Isoforms ROCK1 and ROCK2 Each Contribute to the Development of Experimental Pulmonary Fibrosis. Am. J. Respir. Cell Mol. Biol..

[39] Yin J.-Z., Li Z.-Q., Zhang X.-D., Wan Z.-J., Qin H.-R., Yao L.-H., Li B.-L., Gao F., Yang Y.-Y. (2024). Bufotalin Attenuates Pulmonary Fibrosis via Inhibiting Akt/GSK-3β/β-Catenin Signaling Pathway. Eur. J. Pharmacol..

[40] Henderson N.C., Arnold T.D., Katamura Y., Giacomini M.M., Rodriguez J.D., McCarty J.H., Pellicoro A., Raschperger E., Betsholtz C., Ruminski P.G. (2013). Targeting of Av Integrin Identifies a Core Molecular Pathway That Regulates Fibrosis in Several Organs. Nat. Med..

[41] Bagnato G.L., Irrera N., Pizzino G., Santoro D., Roberts W.N., Bagnato G., Pallio G., Vaccaro M., Squadrito F., Saitta A. (2018). Dual Avβ3 and Avβ5 Blockade Attenuates Fibrotic and Vascular Alterations in a Murine Model of Systemic Sclerosis. Clin. Sci..

[42] Patsenker E., Popov Y., Stickel F., Jonczyk A., Goodman S.L., Schuppan D. (2008). Inhibition of Integrin Alphavbeta6 on Cholangiocytes Blocks Tgfbeta Activation and Retards Biliary Fibrosis Progression. Gastroenterology.

[43] Wu Q., Chen P., Li J., Lin Z., Zhang Q., Kwok H.F. (2023). Inhibition of Bladder Cancer Growth with Homoharringtonine by Inactivating Integrin A5/Β1-FAK/Src Axis: A Novel Strategy for Drug Application. Pharmacol. Res..

