# Supplementary material for: Transcriptome-Guided Drug Repurposing Identifies Homoharringtonine (HHT) as a Candidate for Radiation-Induced Pulmonary Fibrosis
Source: Pharmaceutics. 2025 Dec 18;17(12):1626. doi: 10.3390/pharmaceutics17121626 (PMC12736785; doi:10.3390/pharmaceutics17121626)
Supplement: Supplementary file 1 [file pharmaceutics-17-01626-s001.zip › pharmaceutics-3940388-supplementary.pdf]

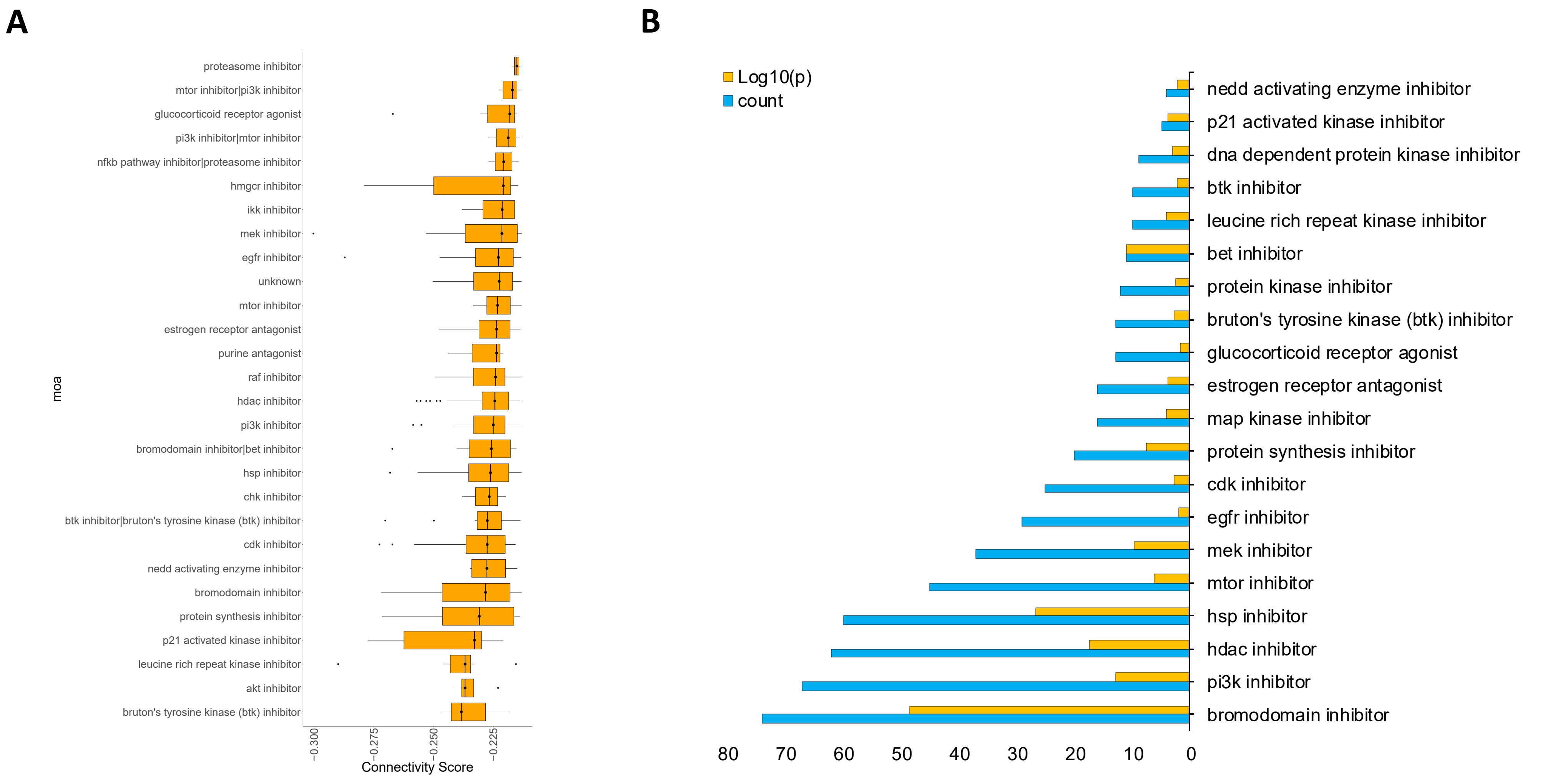

Figure S1. Molecular signature of inflammation process. (A) boxplot representing the transcriptome-reversing matches. (B) MoA-wise analysis comprising count (blue bar) and P value (orang bar).

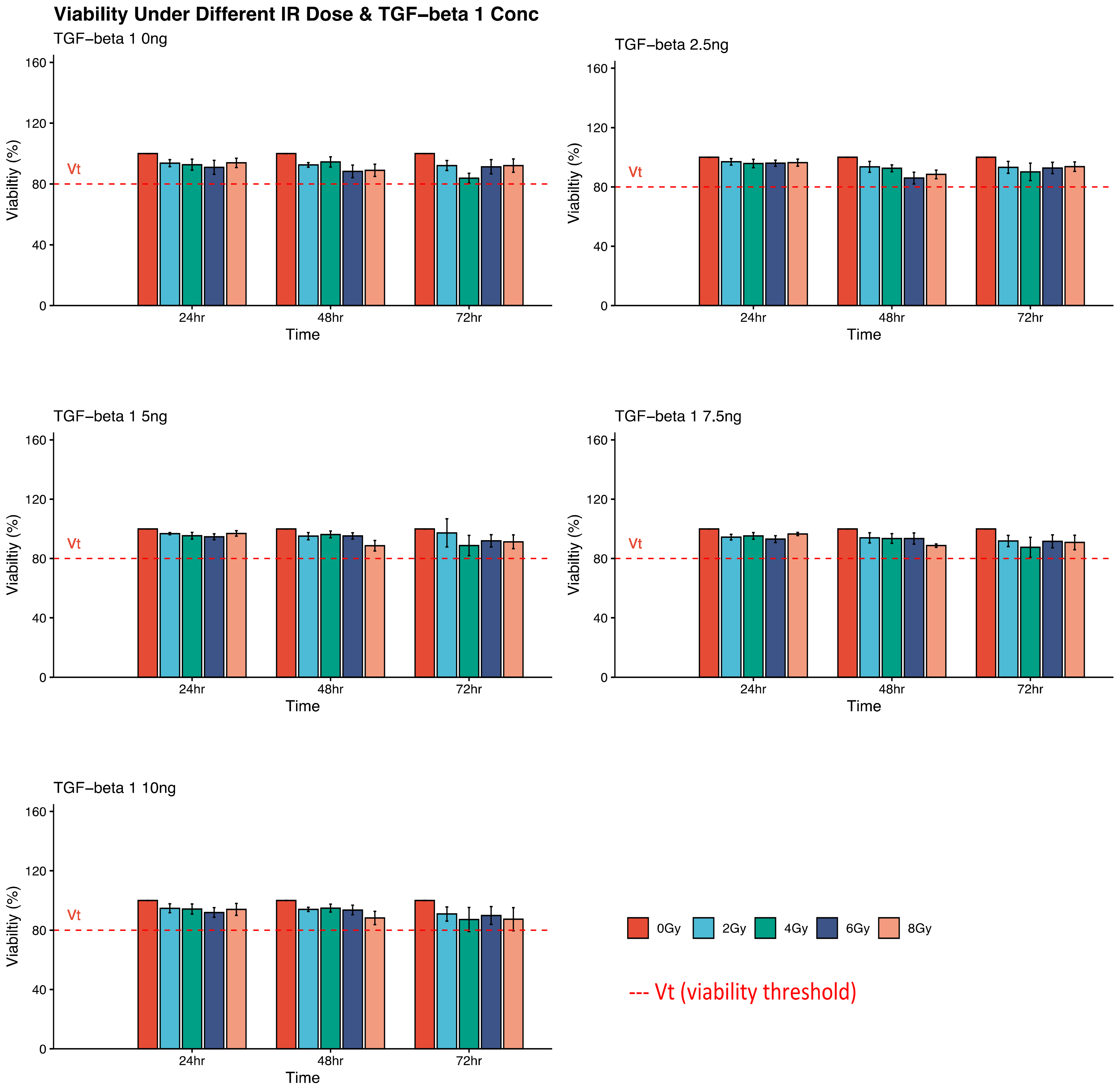

Figure S2. Viability assay of MRC-5 using WST-1 assay after being exposed to different IR doses followed by addition of different concentrations of TGFB1 at 24, 48, 72hr. Viability is considered affected by the treatment when it is below 80%.

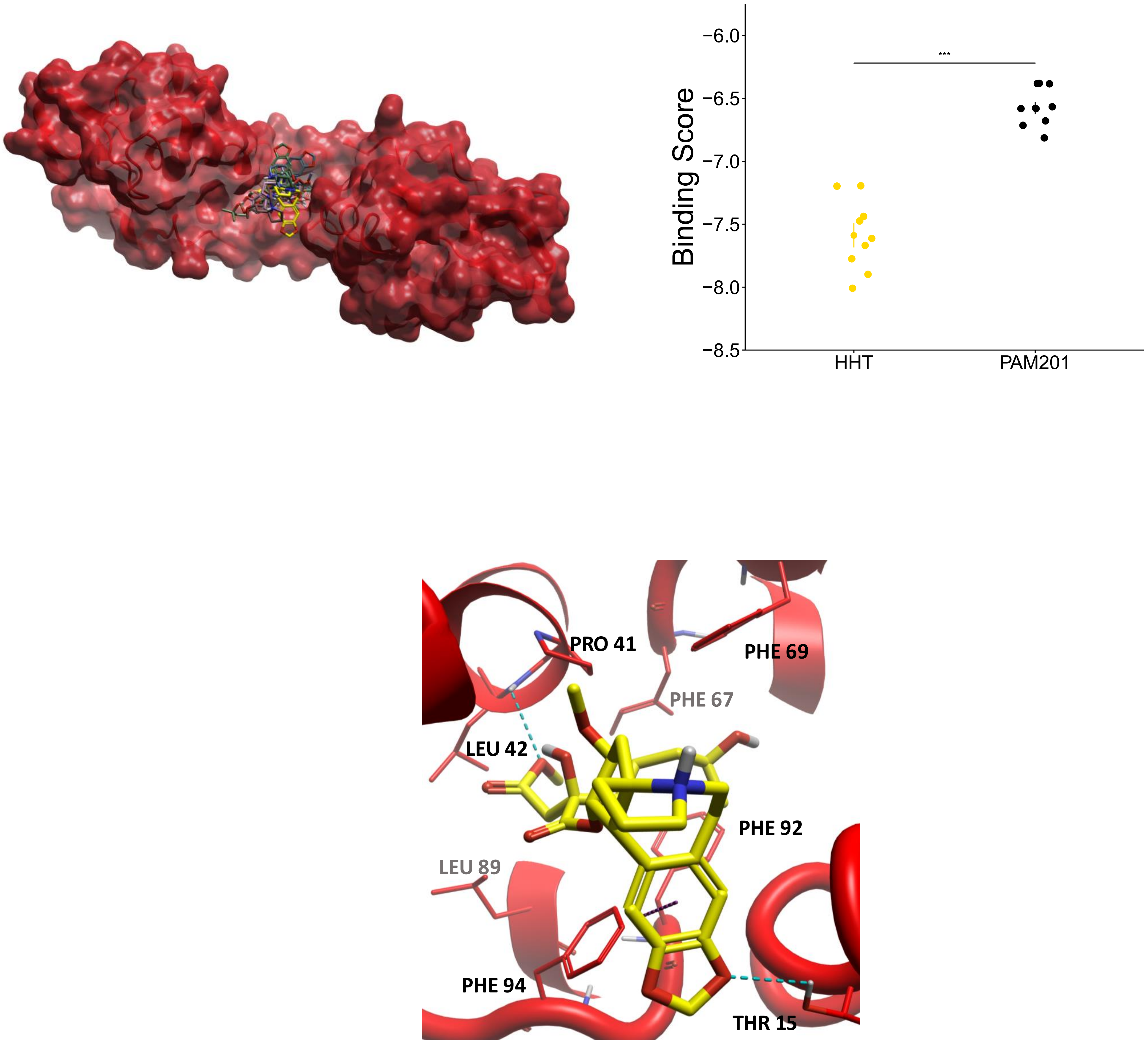

Figure S3. Molecular docking analysis of HHT against homodimeric frizzled receptor showing interacting of HHT with wnt-binding domain.
